# Large-Scale Domain Motions and Pyridoxal-5′-Phosphate Assisted Radical Catalysis in Coenzyme B_12_-Dependent Aminomutases†

**DOI:** 10.3390/ijms15023064

**Published:** 2014-02-20

**Authors:** Amarendra Nath Maity, Yung-Han Chen, Shyue-Chu Ke

**Affiliations:** Physics Department, National Dong Hwa University, Hualien 97401, Taiwan; E-Mails: anmaity@gmail.com (A.N.M.); d9614002@ems.ndhu.edu.tw (Y.-H.C.)

**Keywords:** coenzyme B_12_ (5′-deoxyadenosylcobalamin; dAdoCbl), pyridoxal-5′-phosphate (PLP; vitamin B_6_), lysine 5,6-aminomutase, ornithine 4,5-aminomutase, isotope-edited electron paramagnetic resonance (EPR) and electron-nuclear double resonance (ENDOR) spectroscopy, density functional theory (DFT)

## Abstract

Lysine 5,6-aminomutase (5,6-LAM) and ornithine 4,5-aminomutase (4,5-OAM) are two of the rare enzymes that use assistance of two vitamins as cofactors. These enzymes employ radical generating capability of coenzyme B_12_ (5′-deoxyadenosylcobalamin, dAdoCbl) and ability of pyridoxal-5′-phosphate (PLP, vitamin B_6_) to stabilize high-energy intermediates for performing challenging 1,2-amino rearrangements between adjacent carbons. A large-scale domain movement is required for interconversion between the catalytically inactive open form and the catalytically active closed form. In spite of all the similarities, these enzymes differ in substrate specificities. 4,5-OAM is highly specific for d-ornithine as a substrate while 5,6-LAM can accept d-lysine and l-β-lysine. This review focuses on recent computational, spectroscopic and structural studies of these enzymes and their implications on the related enzymes. Additionally, we also discuss the potential biosynthetic application of 5,6-LAM.

## 1. Introduction

**Scheme 1.**
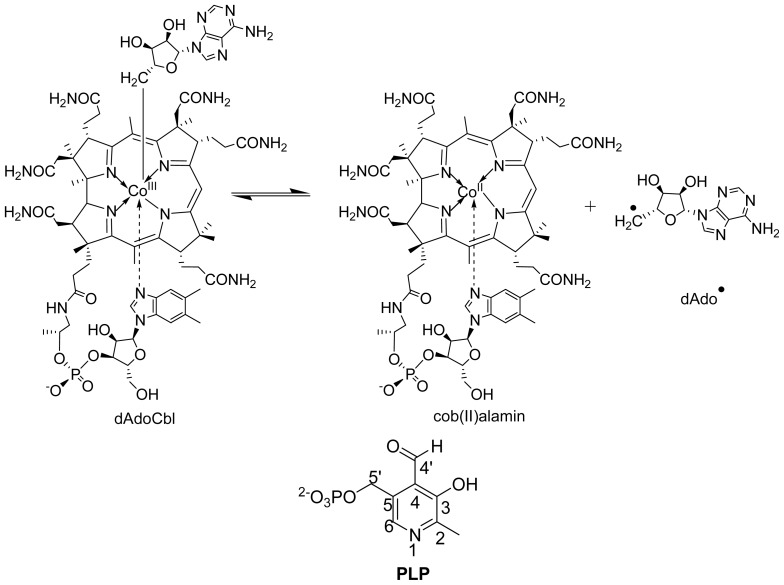
Structures of dAdoCbl, cob (II) alamin, dAdo^•^ and pyridoxal-5′-phosphate (PLP).

Vitamins B_12_ and B_6_ are two well known vitamins that have been studied extensively [1–7]. Coenzyme B_12_ (5′-deoxyadenosylcobalamin, dAdoCbl) and pyridoxal-5′-phosphate (PLP) (Scheme 1), derivatives of vitamin B_12_ and vitamin B_6_, respectively, act as cofactors in numerous enzymes and have attracted enormous interests of both experimentalists and theoreticians [8–23]. dAdoCbl is often described as nature’s “free radical reservoir” because of its ability to produce highly reactive 5′-deoxyadenosyl (dAdo^•^) radical in enzymatic environments. Although in the literature coenzyme B_12_ and 5′-deoxyadenosyl radical have been abbreviated as AdoCbl and Ado^•^ or Ado-CH_2_^•^, respectively, in a recent article [19] Bucher *et al.*, used the more representative, dAdoCbl and dAdo^•^ and we shall follow these notations in this review. PLP plays an important role to stabilize high energy intermediates in enzymes.

**Scheme 2.**
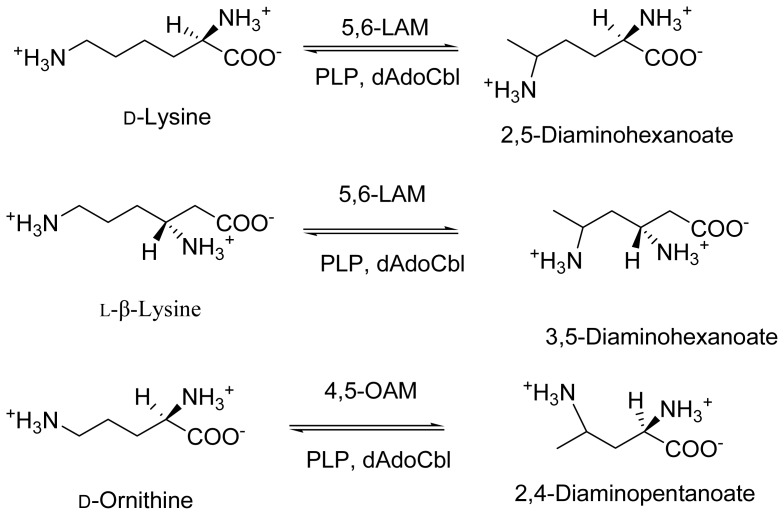
Reactions of lysine 5,6-aminomutase (5,6-LAM) and ornithine 4,5-aminomutase (4,5-OAM).

Lysine 5,6-aminomutase (5,6-LAM; EC 5.4.3.4) and ornithine 4,5-aminomutase (4,5-OAM; EC 5.4.3.5) are two of the rare enzymes that use both dAdoCbl and PLP as cofactors. They belong to a Class III dAdoCbl-dependent isomerase family. 5,6-LAM catalyzes the reversible interconversion of d-lysine and 2,5-diaminohexanoic acid, and l-β-lysine and 3,5-diaminohexanoic acid, whereas 4,5-OAM catalyzes reversible interconversion of d-ornithine and 2,4-diaminopentanoic acid (Scheme 2). Some aspects of 5,6-LAM along with lysine 2,3-aminomutase (2,3-LAM), a related enzyme that uses PLP and *S*-adenosyl-l-methionine (SAM) as cofactors to catalyze interconversion of l-lysine and l-β-lysine, were recently reviewed by Frey and Reed [24] and some of the structural aspects of 5,6-LAM and 4,5-OAM for controlling radical chemistry have been discussed by Drennan *et al.* [25]. In this review we present a comprehensive and comparative account of all aspects, with an emphasis on recent observations of 5,6-LAM and 4,5-OAM, which are very similar yet different.

## 2. Discovery and Physiological Role

Lysine and ornithine are degraded in a similar manner in *Clostridium sticklandii,* a Gram-positive anaerobe. 5,6-LAM participates in the second step of the fermentation pathway of lysine in which lysine is converted to acetic acid, ammonia and butyric acid, while 4,5-OAM takes part in the first step of the fermentation pathway of ornithine, in which ornithine is converted to acetate, carbon dioxide, alanine and ammonia by clostridia [26]. Dr. Theressa Stadtman and coworkers first discovered and performed initial studies on 5,6-LAM [26,27]. Initially, it was assumed that there are two distinct enzymes [28–31] displaying 5,6-aminomuatase activity relating to two distinct substrates, d-lysine and l-β-lysine. Later, it was found that these are the same enzyme, which can accept two different substrates [26,32]. Dyer and Costilow [33] reported OAM activity for the first time in crude extracts, and a subsequent study by Tsuda and Friedmann on cofactor requirements was performed using partially purified extracts [34]. Later, separation, purification and properties of 4,5-OAM was reported by Somack and Costilow [35].

5,6-LAM comprises two protein components, the core enzyme E_1_ and an auxiliary activating protein E_2_. E_1_ is a 170 kDa heterotetramer composed of α- (55 kDa) and β- (30 kDa) subunits and formulated as α_2_β_2_, whereas the molecular mass of E_2_ was estimated to be ~80 kDa. E_2_, which showed dAdoCbl synthetase activity when isolated separately, could activate and transfer radioactivity from [8-^14^C]ATP to E_1_ [31]. In addition, the presence of E_2_ in the assay mixture induces ATP to activate E_1_ allosterically. 4,5-OAM is also a α_2_β_2_ heterodimer. The molecular mass of 4,5-OAM, which comprises two strongly associating subunits having molecular masses of 12.8 and 90.0 kDa, was estimated to be about 200 kDa [26].

## 3. Cloning and Expression of Recombinant Aminomutases

There was absence of research for almost three decades after the initial studies on both of these enzymes as degraded forms of cobalamin often remain tightly bound to the enzymes purified from clostridia. Taking advantage of recombinant technology and keeping in mind that *E. coli* does not synthesize cobalamins *de novo*, Chang and Frey solved the problem in the year 2000 by cloning the genes encoding the α- and β-subunits of 5,6-LAM in *C. sticklandii*, co-expressing them in *Escherichia coli* and subsequent purification to obtain cobalamin-depleted 5,6-LAM [36]. The recombinant enzyme (KamDE) containing only E_1_ was found to be active. However, it was subjected to suicide inactivation with the substrate [23,37]. The large α subunit contains 538 residues, whereas the small β subunit contains 262 residues. Later, nearly identical genes were cloned from *Porphyromonas gingivalis*, expressed similarly, and the produced protein was purified [38]. Chen *et al.*, reported cloning of *OraS* and *OraE*, the genes encoding components *OraS* and *OraE* of 4,5-OAM from *C. sticklandii*, followed by heterologous expression in *E. coli*, and purification of recombinant proteins [39]. OraS (12.8 kDa) contains 121 amino acid residues while OraE contains 753 amino acid residues. The molecular mass of about 201 kDa of recombinant 4,5-OAM suggests that it is an α_2_β_2_ heterodimer. The coexpression, purification and characterization of *OraS* and *OraE* was reported along with mutant protein, OraSE-K629M, which proves that Lys629 is responsible for the binding of PLP [40].

Although ATP was found to be an allosteric regulator for 5,6-LAM [28], the recombinant 5,6-LAM does not possess ATP-dependent allosteric activity [36]. Interestingly, OraS of 4,5-OAM is capable of forming a complex with KamDE of 5,6-LAM and restores the allosteric regulation of ATP [41].

The easy access to the recombinant enzymes facilitated subsequent studies to unravel the structure and mechanism of action that are discussed in the following sections. The kinetic properties of the recombinant enzymes with respective substrates are summarized in Table 1.

## 4. Structural Studies

**Figure 1.**
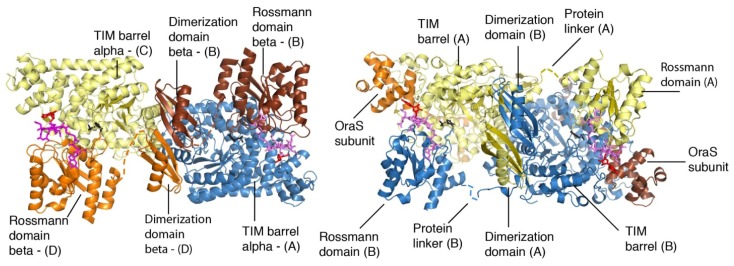
Crystal structures of (**left**) 5,6-LAM and (**right**) 4,5-OAM in open state (Adapted with permission from reference [25]. Copyright 2012 Annual Reviews).

dAdoCbl-dependent mutases utilize the ubiquitous triosephosphate isomerase (TIM) barrel fold and the common Rossmann fold to manage radical chemistry [25]. Berkovitch *et al.*, reported the crystal structure of the substrate-free holoenzyme form of 5,6-LAM [44]. dAdoCbl is situated in the Rossmann domain in the crystal structure, and separated from PLP, which is bound at the top of the TIM barrel domain, by a distance of ~25 Å (Figure 1). The structure further reveals that the Rossmann domain is tilted toward the edge of the TIM barrel; this conformation is termed as “edge on” conformation or open state. This is in contrast to a “top on” conformation or a closed state, in which the Rossmann domain is located directly above the TIM barrel, observed in the substrate bound structures of methylmalonyl-coenzyme A mutase (MCM) and glutamate mutase (GM) [25]. This suggests the requirement of a large scale conformational change, in which the “edge on” conformation or open state reversibly transforms to “top on” conformation or closed state to achieve the catalysis. Several crystal structures of 4,5-OAM, including the substrate-free resting state, were reported by Wolthers *et al.* [45]. The crystal structure of substrate-free 4,5-OAM is similar to that of 5,6-LAM and a separation of ~ 23 Å between dAdoCbl and PLP was observed in the resting state (Figure 1). The study also indicates that 4,5-OAM can assume a closed state within the confines of the crystal lattice.

### 4.1. Pyridoxal-5′-Phosphate (PLP) Binding Site and Active Site Residues

**Figure 2.**
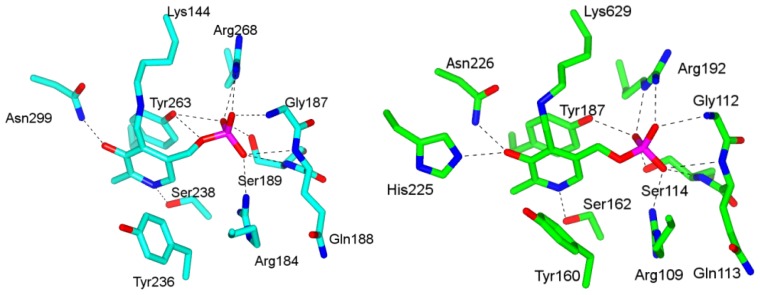
Binding sites of PLP in (**left**) 5,6-LAM (PDB accession code: 1XRS) and (**right**) 4,5-OAM (PDB accession code: 3KP1). These figures were generated using CCP4MG Version 2.7.3.

**Figure 3.**
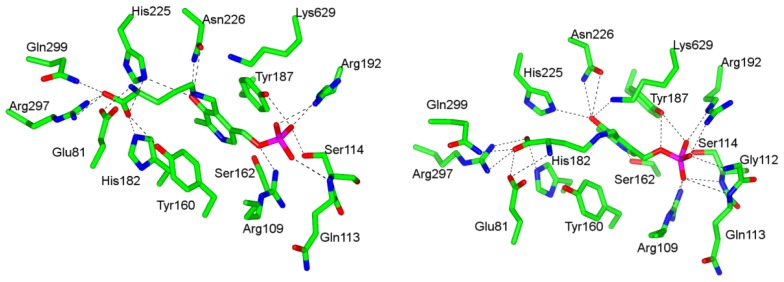
Binding sites of (**left**) PLP-ornithine adduct (PDB accession code: 3KOZ) and (**right**) PLP-DAB adduct (PDB accession code: 3KOX) in 4,5-OAM. These figures were generated using CCP4MG Version 2.7.3.

5,6-LAM shares features with PLP-dependent enzymes of fold types II, III and IV [46]. Like fold type III enzymes, PLP is situated in the TIM barrel. Nevertheless, the PLP forms imine linkage with Lys144, which belongs to the Rossman domain rather than the TIM barrel. The presence of a serine residue, Ser238, in 5,6-LAM [44] for hydrogen-bonding interaction with pyridine nitrogen of PLP is similar to fold type II enzymes. Solvent exposed *si* face of PLP, the feature of fold types III and IV, is also observed in 5,6-LAM. Other important interactions of PLP with amino acid residues include π-electron stacking interactions with Tyr263, hydrogen bonding of phenolic oxygen with Asn299, and multiple interactions of phosphate group with side chains of Arg184, Arg268, Ser189 and main chain amides of Gly187, Gln188, and Ser189. We also note that Tyr236 makes hydrophobic interaction with PLP (Figure 2). Comparison of PLP binding sites between 5,6-LAM and 4,5-OAM reveals that the PLP-binding interactions are highly conserved between 5,6-LAM and 4,5-OAM [45] with the exception of an additional hydrogen bonding interaction between His225 and PLP phenolic oxygen in 4,5-OAM (Figure 2). Conserved Lys629 forms an imine link with PLP in 4,5-OAM.

d-Ornithine soaked 4,5-OAM crystals [45] provided the glimpse of substrate bound structure, in which Lys629 has moved away from PLP and ornithine is bound to PLP through external aldimine. Hydrogen bonding and salt bridge interactions of Gln299, Arg297, His225, Tyr160, and His182 with carboxylate moiety of d-ornithine and electrostatic interaction between Glu81 and α-amine anchor the substrate in the active site (Figure 3). The hydrogen bonding interaction of pyridine nitrogen with Ser162 is absent. Similar interactions are observed in the case of substrate analog dl-2,4-diaminobutyric acid (2,4-DAB) with few exceptions. His225 and Tyr160 do not make hydrogen bonds with the carboxylate while Ser162 and Lys629 make additional hydrogen bonds with pyridine nitrogen and phenolic oxygen, respectively (Figure 3).

### 4.2. “Base-off”/“His-on” Binding Mode of dAdoCbl

Before the crystal structure could be obtained, “base-off”/“His-on” binding mode of dAdoCbl in 5,6-LAM was confirmed by comparing the electron paramagnetic resonance (EPR) spectra of methylhydrazine treated 5,6-LAM reconstituted with [^15^N-DMB]dAdoCbl, photolyzed natural abundant dAdoCbl in solution, and enriched [^15^N-DMB]dAdoCbl in solution [36]. The observance of triplet superhyperfines originating from ^14^N (*I* = 1) rather than a doublet superhyperfine feature corresponding to ^15^N (*I* = 0.5) [47] suggests that the ^15^N-enriched dimethylbenzimidazole (DMB) ligand has been replaced by the nitrogen atom of a histidine residue. The EPR experiments with ^15^N-labelled protein demonstrated the “base-off”/“His-on” binding mode of dAdoCbl in GM [48]. This is in contrast to the spectra observed in dAdoCbl-dependent enzyme ribonucleoside triphosphate reductase (RTPR), where the experiments with [^15^N-DMB]dAdoCbl identified DMB as the axial ligand [49].

The crystal structures revealed that dAdoCbl binds to 5,6-LAM and 4,5-OAM in the “base-off”/“His-on” mode, in which the DMB is replaced by His133β and His618, respectively. This mode of binding for dAdoCbl has been observed in methionine synthase (MS) [50], MCM [51], and GM [52]. In contrast to GM [53], MCM [54], and free cofactor dAdoCbl [55], where dAdo moiety assumes anti conformation about the glycosidic bond, the dAdo moiety is present in syn conformation in 5,6-LAM and 4,5-OAM.

Unlike in any dAdoCbl dependent enzymes (5,6-LAM [44], MS [50], GM [53], and MCM [51]) Co–C bond is intact in 4,5-OAM. X-ray induced photoreduction of the Co–C bond was reported for dAdoCbl enzymes [56]. The origin of unusually stable Co–C remains, however, unknown yet.

### 4.3. Models of Closed Conformation

**Figure 4.**
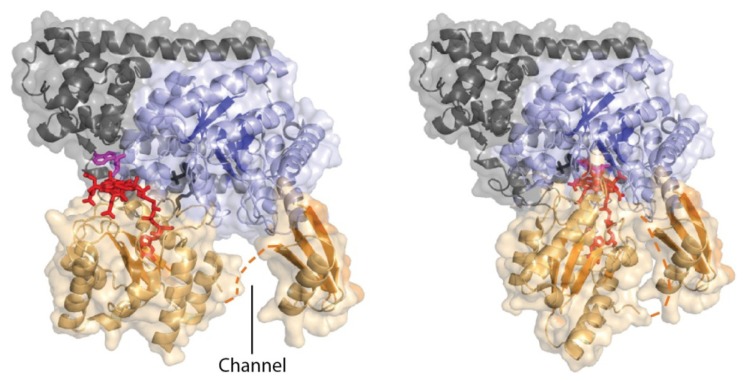

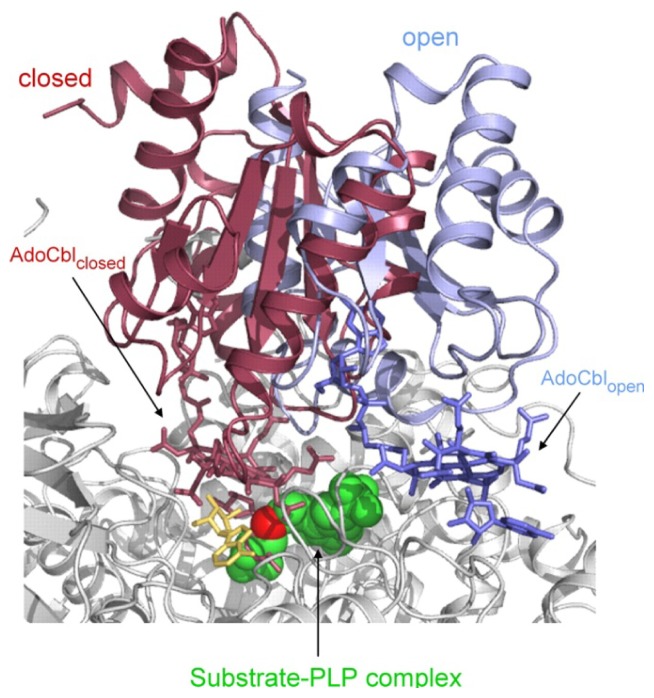
Models of closed conformations of (**top**) 5,6-LAM and (**bottom**) 4,5-OAM (Adapted with permissions from references [25] and [45]. Copyright 2012 Annual Reviews and Copyright 2010 American Society for Biochemistry and Molecular Biology).

The inability to obtain crystal structure of catalytically active closed conformation prompted the researcher to model [25] it by superimposing the Rossmann domains of 5,6-LAM and MCM, while the model of closed conformation of 4,5-OAM [45] was obtained by superimposing with GM (Figure 4). An interesting finding of the model studies is that the enzymes are capable of Rossmann domain swing, which brings dAdoCbl near the active site. In addition, the model of 4,5-OAM reveals that a reorientation of the *syn* and eastern conformation of the adenosine moiety to *anti* and western conformation is required to avoid the direct overlap with the substrate in the active site. Moreover, the reorientation positions the C5′ of dAdo moeity within van der Waals distance to the substrate so that the dAdo^•^ can abstract a hydrogen from the substrate.

## 5. Domain Motions

Large-scale domain motions are often used by proteins to protect transient reaction intermediates [57–61]. Large-scale conformational change upon substrate binding is common in cobalamin dependent enzymes, MS [62,63] and MCM [54]. As mentioned above, the crystal structures of both 5,6-LAM and 4,5-OAM suggest large-scale domain motions are required to achieve catalysis. Although MCM, 5,6-LAM and 4,5-OAM have very similar structures, the manner how the conformational change occurs in MCM differs with that in 5,6-LAM and 4,5-OAM. Two different strategies have been developed by these enzymes to tune with the unique properties of the substrates. Larger substrate methylmalonyl-coenzyme A binds to a large gap in the center of the TIM barrel and causes conformational change facilitating homolysis of Co–C bond, while smaller substrates of 5,6-LAM and 4,5-OAM release an enzyme bound lysine from the PLP resulting in free movement of Rossmann domain and conformational rearrangement. In 4,5-OAM, this proposition was investigated by molecular dynamics (MD) simulations along with quantum mechanical and molecular mechanical (QM/MM) calculations [64]. The MD simulation of the open form was performed based on the substrate bound crystal structure of 4,5-OAM [45], while the closed form was based on the model constructed by superimposing the relevant domains onto those in the structure of GM, as described above (see Section 4.3).

**Figure 5.**
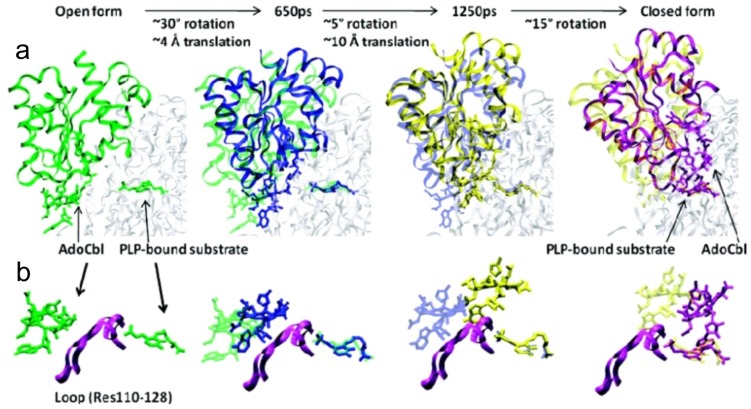
Atomic description of the conformational rearrangement of the Rossmann domain. (**a**) Snapshots along the trajectory 1 (with the targeted molecular dynamics (TMD) simulation time labeled) are selected to highlight the rotational and translational movement; and (**b**) Close-up of the cobalamin ring and PLP-bound substrate within each snapshot in top. The loop formed by residues 110–128 that “gates” the active-site cavity is displayed in purple ribbon (Adapted with permission from reference [64]. Copyright 2012 American Chemical Society).

The results suggest that a combination of a ~52° rotation and a ~14 Å translation is needed to bring dAdoCbl into the active site cavity [64]. The snapshots along one of the trajectories are shown in Figure 5. Initially, a ~30° rotation of the Rossmann domain is required to bring dAdoCbl to the proximity of active site. The impediment, to access the active site, originating from a loop region (residues 110–128) of the TIM barrel is overcome by a sequential translation-rotation: first a translation of ~12–14 Å with a subsequent ~15°–20° rotational movement of Rossmann domain. Targeted molecular dynamics (TMD) simulations indicate that the conformational change from eastern to the northern of dAdo moiety takes place at the later stage of the Rossmann domain conformational rearrangement. During this stage, it predominantly involves rotational movement to facilitate dAdo conformational change, in which it interacts favorably with the active site residues. For example, Glu338, a residue that is more than 20 Å away in the open form, forms hydrogen bonding interactions with the hydroxyl groups of the ribose ring of dAdo moiety. These rotational and translational movements are found to be concerted when it moves from closed form to open form.

## 6. Mechanistic Studies

**Scheme 3.**
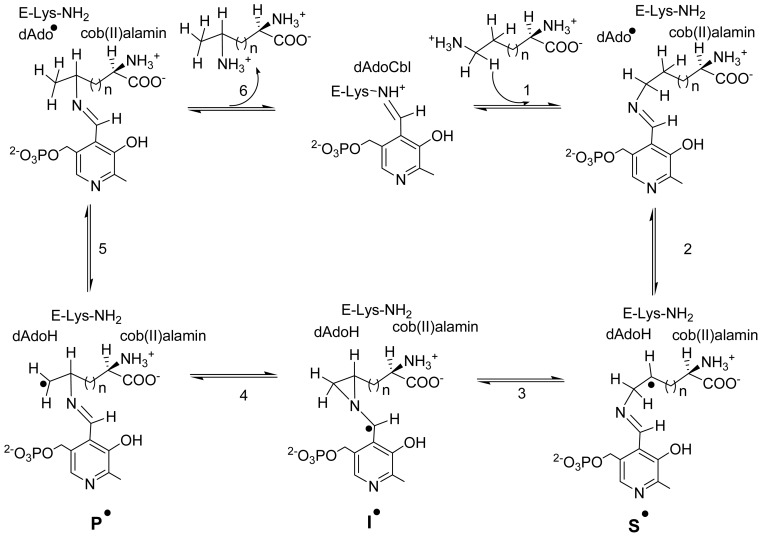
Proposed mechanism of 5,6-LAM (*n* = 2) and 4,5-OAM (*n* = 1).

The chemical mechanism for both the enzymes was proposed [32] using an analogy from the related enzyme 2,3-LAM, in which the PLP assisted radical mechanism had already been established [65–67], and is depicted in Scheme 3. Moreover, a chemical model supporting this rearrangement reaction was also reported [68]. However, the radical generation in 2,3-LAM is achieved in a different manner than 5,6-LAM and 4,5-OAM, as the cofactors are different.

In this mechanism, binding of substrates followed by formation of external aldimines with PLP replacing the internal aldimine with respective lysine residue begins the catalysis. In the mean time, a large scale domain movement brings the dAdoCbl near the substrate-PLP and initiates Co–C bond cleavage giving rise to dAdo^•^ and cob (II) alamin. Next, dAdo^•^ abstracts a hydrogen atom from C5 (5,6-LAM) or C4 (4,5-OAM) of the substrate to produce substrate related radical (S^•^) and 5′-deoxyadenosine (dAdoH). Intramolecular rearrangement of S^•^ through an azacyclopropylcarbinyl intermediate radical (I^•^) generates product related radical (P^•^). In a subsequent step, P^•^ takes back a hydrogen atom from dAdoH to give rise PLP-product and dAdo^•^, which combines with cob (II) alamin to generate dAdoCbl. In the mean time, the lysine residue (144 for 5,6-LAM or 629 for 4,5-OAM) forms internal aldimine with PLP again to release the product and makes it ready for the next catalytic cycle. Therefore, one role of PLP is to send a signal through transaldimination to initiate a large conformational change and eventually cleavage or formation of a Co–C bond. The evidence for most of the steps of this mechanism have been acquired by UV-visible, EPR, and electron nuclear double resonance (ENDOR) spectroscopy. Explanations of these observations as well as propositions for future observations have been documented with the help of high-level computational studies.

### 6.1. UV-Visible Spectroscopic Studies

The changes observed in UV-visible spectra of the enzymes have been used to gain insight about tarnsaldimination and the state of dAdoCbl.

#### 6.1.1. Spectral Changes Originating from PLP

The characteristic band for PLP–Lys144β internal aldimine in 5,6-LAM is observed at 423 nm. Addition of substrate or analogue causes decrease in the absorbance at 423 nm and slight redshifts to 432 nm due to formation of external aldimine with the substrate or analogue [69]. In the case of 4,5-OAM, a broad absorbance shoulder at 416 nm corresponds to internal aldimine, which transforms to external aldimine with ornithine and gives rise to an absorbance peak at 425 along with decrease in the absorbance shoulder at 416 nm [70].

#### 6.1.2. Spectral Changes Originating from dAdoCbl

The absorbance peaks at 335, 378 and 528 nm correspond to dAdoCbl in holo-4,5-OAM [70]. A small decrease of absorbance at 528 nm confirms Co–C bond homolysis upon substrate binding. Steady-state accumulation of cob (II) alamin, indicated by absorbance peak at 470 nm, has not been observed with the substrate due to the instability of ornithiyl-PLP derived radical intermediates. On the contrary, a significant decrease of absorbance at 528 nm and simultaneous appearance of 470 nm was observed with the inhibitor, dl-2,4-diaminobutyric acid [70].

The suicide inactivation of 5,6-LAM is associated with generation of cob (III) alamin through an electron transfer from cob (II) alamin to PLP-substrate/product radical intermediate and indicated by the increase in absorbance of the peak at 358 nm [37]. Substrate induced inactivation has precedent in other dAdoCbl-dependent MCM [71] and DDH [72]. 4,5-OAM also undergoes suicide inactivation, albeit at a rate 10-fold lower than 5,6-LAM [73].

### 6.2. Stopped-Flow Analysis

Stopped-flow absorbance changes have also been employed for pre-steady-state kinetic analysis for 4,5-OAM [70]. Comparison of the UV-Vis spectra of wild type 4,5-OAM with H225Q and H225A mutants revealed the probable role of His225 in stabilizing the radical intermediates [73]. Stopped-flow analysis of the 4,5-OAM variants, E338Q, E338D, and E338A, showed that the conserved glutamate residue plays an important role in controlling the Co–C bond homolysis [43]. Similar interaction of ribose 2′-OH and 3′-OH with conserved Glu330 in glutamate mutase was suggested for dAdo^•^ stabilization [74].

### 6.3. Electron Paramagnetic Resonance (EPR) and Electron Nuclear Double Resonance (ENDOR) Spectroscopy

EPR and ENDOR spectroscopy have been the principal tools to unravel radical intermediates, including magnetically coupled species [75] in enzymatic systems [76–79]. EPR spectroscopy of B_12_-dependent enzymes were documented in chapters of two books [80,81]. Following the trend, the EPR and ENDOR spectroscopy have been employed for both the enzymes.

#### 6.3.1. Distance between PLP-Substrate/Analogue Radical and Cob (II) Alamin

**Scheme 4.**
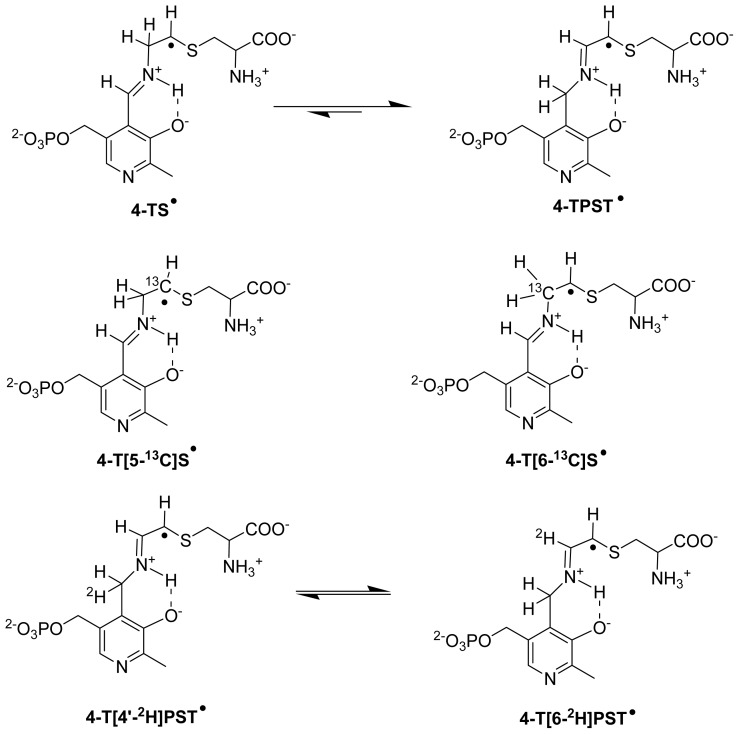
Natural abundance and isotope-edited transient (4-TS^•^) and persistent radical (4-TPST^•^) structures.

The positions of radical intermediates in the active sites were reviewed by Reed and Mansoorabadi [82]. The first direct proof of radical mechanism in these enzymes was obtained in the reaction of the substrate homologue 2,4-DAB with 4,5-OAM only five years back [70]. A distance of less than 6 Å was estimated between the two paramagnetic species for 4,5-OAM similar to that reported for glutamate mutase [83] and MCM [84]. Nevertheless, two unlikely structures initially proposed by authors were dismissed and a highly stabilized radical centered at C4 of 2,4-DAB was proposed [85]. This proposition found support in our homologue based study [69] in the reaction of 5,6-LAM which was complemented by theoretical calculations (see below). In the case of 5,6-LAM, the evidence for the radical mechanism was provided by the observation of transient (4-TS^•^, Scheme 4) and persistent radical (4-TPST^•^, Scheme 4) in the reaction of 4-thia-l-lysine with 5,6-LAM [85,86]. The distance between Co^2+^ and transient and persistent radicals were estimated as ~7 and ~10 Å, respectively [85]. The structures of transient and persistent radicals were obtained by isotope-edited EPR and ENDOR spectroscopy (see below). A weaker spin-spin interaction was observed in the signal of a substrate radical intermediate in ethanolamine ammonia-lyase (EAL) [87–89] and diol dehydrase (DDH) [90].

#### 6.3.2. Isotope-Edited EPR and ENDOR Spectroscopy for Characterization of Radical Intermediates

Two studies with isotope-edited PLP [86] and substrate analogues [85] indicated the corresponding structures of transient and persistent radical. The observation of isotropic hyperfine couplings from deuterium (^2^H), in addition to ^31^P, in ENDOR measurements in the reaction of [4′-^2^H]PLP (Scheme 4) confirms that the persistent radical is PLP-substrate based species while EPR measurements with ^13^C, ^2^H, and ^15^N labeled 4-thia-l-lysine suggested both the transient and persistent radicals are 4-thia-l-lysine based species.

To make unequivocal characterization of the radical intermediates, we synthesized site-directed isotopomers, 4-thia-[5-^13^C]lysine [91] and 4-thia-[6-^13^C]lysine [92], and performed EPR experiments [93]. The line broadening observed for 4-thia-[5-^13^C]lysine derived transient radical (4-T[5-^13^C]S^•^, Scheme 4) and absence of that for 4-thia-[6-^13^C]lysine derived transient radical (4-T[6-^13^C]S^•^, Scheme 4), confirmed that the radical is centered on C5 of 4-thia-l-lysine. Evidently, the transient radical is the corresponding 4-thia analogue of S^•^ (4-TS^•^, Scheme 4). Expected line broadenings were also observed with both 4-thia-[5-^13^C]lysine and 4-thia-[6-^13^C]lysine in the case of persistent radical (4-TPST^•^, Scheme 4) as C6 2p orbital overlaps with the C5 spin orbital and induces 6-^13^C line broadening in addition to 5-^13^C line broadening.

#### 6.3.3. Role of Conserved Tyr Residue

From the crystal structures of both 5,6-LAM and 4,5-OAM, it is evident that a conserved tyrosine residue is interacting with PLP through π-stacking interaction. The role of this conserved tyrosine was investigated in 5,6-LAM by means of site-directed mutagenesis of Tyr263 and EPR experiments [93]. In the early reaction (<10 s) with 4-thia-l-lysine, the Y263F mutant produced identical signal as wild-type 5,6-LAM. With prolonged reaction time, the Y263F mutant produced a different spectra than wild-type 5,6-LAM. The spectra reveal three components—a spin-coupled component, which is similar to that of persistent radical (4-TPST^•^) of wild-type 5,6-LAM, centered at *g* = 2.112, a cob (II) alamin component with *g*^⊥^ = 2.34 and a new radical intermediate component at *g* = 2.006. This means that the spin-coupled component is unstable and gives rise to the other two species. It was shown that Tyr263 does not come into play until spin is delocalized or formation of 4-TPST^•^. Another interesting observation was that the peak at *g* = 2.006 was not present when reacted under aerobic condition while the persistent radical is not acutely sensitive to air. Taken together, these results suggest that Tyr263 stabilizes the persistent radical as well as prevents it from oxidative damage. Moreover, this indicates that Tyr263 might play a role in stabilizing the azacyclopropylcarbinyl radical (I^•^, Scheme 3) by either modulating the spin distribution via stacking interaction or locking it in particular conformation.

#### 6.3.4. Transition between Open and Closed State

Using substrate homologues, spin-coupled and uncoupled spectra were obtained suggesting the presence of closed and open state in the case of 5,6-LAM [69]. It was found that the homologues induce Co–C bond homolysis. In the case of d-2,5-diaminopentanoic acid (2,5-DAPn) and 2,4-DAB, dAdo^•^ abstracts hydrogen atoms from the carbon adjacent to the imine and leads to highly stabilized radicals by spin delocalization into the pyridine ring of PLP. These overstabilized radicals, which are unable to take back the hydrogen atom from dAdoH, block the active site and inhibit the enzyme. Another interesting finding of the study was that for analogue containing even numbered of carbon atoms, 4-thia-l-lysine (4-*S*-2,6-DAH) and 2,4-DAB, cob (II) alamin is spin coupled with PLP-analogue based radicals while odd numbered carbon containing analogues, 2,5-DAPn and d-2,3-diaminopropanoic (2,3-DAPr) acids, produce uncoupled spectra. Spin-coupled spectra correspond to a closed state while the uncoupled spectra indicate the transition to an open state. Therefore, these results provide the proof for transition between open and closed state during catalysis.

### 6.4. Computational Studies

**Scheme 5.**
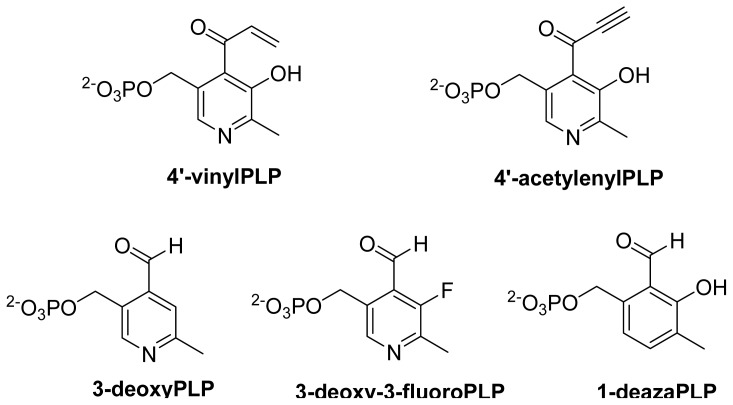
Molecular structures of PLP analogs.

Computational studies, DFT in particular, have been of immense help to unravel the radical mechanism in enzymes. Radom and coworkers have published two articles, which provide the theoretical support for the radical isomerization based mechanism [94,95] in these enzymes. In these studies, by comparing relative energies calculated at RMP2/G3MP2Large of B3LYP/6-31G(d,p) optimized structutres, three important roles of PLP were suggested: first, introduction of double bond into the migrating group; second, stabilization of I^•^ by spin delocalization into the pyridine ring; third, prevention of overstabilization of I^•^ through hydrogen bonding interaction of hydroxyl group. Later using similar methodology, they explained [96] the observation of substrate based radical intermediates [65,67,97] in the case of 2,3-LAM and reported that the reaction of 4-thia-l-lysine would also provide the detection of 4-TS^•^ in the reaction with 5,6-LAM. The azacyclopropylcarbinyl radical (I^•^), the missing link, was not detected in enzymatic systems. It was observed by EPR at temperature less than −130 °C [98]. The calculation with the models suggest that 4′-vinylPLP and 4′-acetylenylPLP (Scheme 5), if accepted by enzyme, would accumulate corresponding I^•^ in the reaction of 5,6-LAM [96]. Though the authors did not mention, we note here that it should also be the case in 4,5-OAM. Nevertheless, it might be more difficult for 4,5-OAM to accommodate the bulky PLP analogue than 5,6-LAM as 4,5-OAM is more substrate-specific than 5,6-LAM. The authors also suggested that the corresponding P^•^ could be detected by EPR in the reaction of 5,6-LAM with either *cis*-2,5-diaminohex-4-enoate or *cis*-3,5-diaminohex-4-enoate. Following the procedure established by Radom and coworkers, DFT have been efficiently employed to unravel the structure of radicals by complementing the experimental results. For instance, relative energies of optimized structures obtained using B3LYP/6-311+G(d,p) and comparison of hyperfine coupling constants (HFCCs) of the ^2^H obtained using B3LYP/TZVP with ENDOR data excluded four out of five proposed candidates [85] for persistent radical in the reaction of 4-thia-l-lysine with 5,6-LAM.

Later, spin delocalization into the pyridine ring in the case of I^•^ is illustrated using spin density distribution obtained at the level of B3LYP/6-31G(d,p) to explain the possible role of Tyr263 in stabilizing the I^•^ through π-stacking interactions [93]. Very recently, the comparison of HFCCs of ^2^H and ^31^P obtained from ENDOR and calculated for various plausible structures of the homologue-based radicals reveals that the values are in agreement with the radical centered at the carbon adjacent to imine [69]. With the help of DFT calculation the probable role of relative orientations at C2 of the substrate in 5,6-LAM for the transition between open and closed state was also suggested [69].

We have recently investigated the case of 5-fluorolysine as an alternative substrate in the reaction of 5,6-LAM and found that the substrate related radical (5-FS^•^) could be detected [99]. In addition, we prepared an inventory of ^19^F HFCCs, originating from significant amount of spin density on fluorine nucleus (*I* = 0.5), using various relevant combinations of methods, MP2 and DFT, and basis sets. This would help to make the right choice of method and/or basis set for calculating ^19^F HFCCs, which is very challenging to predict, of similar systems.

## 7. Outlook and Perspectives

A substantial amount of information has been obtained from the studies on both 5,6-LAM and 4,5-OAM. The recent independent studies on these enzymes are, in some sense, almost complementary to each other. The studies on 5,6-LAM concentrated more on unraveling the radical mechanism while the studies on 4,5-OAM paid attention to domain motion as well as role of specific residues. An exchange of these studies might yield interesting observations. In spite of these findings, many questions remain unanswered. Further studies are needed to address those questions. Below we discuss some probable studies that can be performed in the future to gain further insight into these enzymes.

By comparing the crystal structures of substrate bound 4,5-OAM and substrate free 4,5-OAM and making an analogy in the case of 5,6-LAM it would be possible to identify the residues responsible for subatrate binding in 5,6-LAM, and subsequent mutagenesis would help to confirm the identification. For example, mutation of Asp298 to histidine in 5,6-LAM would be interesting as Asp298 in 5,6-LAM closely overlays with His225 of 4,5-OAM. His225 might play a role to make 4,5-OAM more substrate specific than 5,6-LAM. So, studies on the His225Asp mutant of 4,5-OAM might shed some light on that too. With the availability of this information, one would be able to modulate the active site according to substrate analogue by performing meaningful mutagenesis.

By careful investigation of the crystal structure and amino acid sequence we have identified Glu341 as the conserved glutamate residue potentially responsible for stabilizing dAdo^•^ in 5,6-LAM. Mutagenesis, like in 4,5-OAM [43], of Glu341 in 5,6-LAM would provide information regarding the involvement of it in the catalysis. Similarly, mutagenesis studies of conserved tyrosine in 4,5-OAM as performed in the case of 5,6-LAM would be interesting. The role of conserved serine, which makes a hydrogen bonding interaction with pyridine nitrogen, remains to be investigated in both 5,6-LAM (Ser238α) and 4,5-OAM (Ser162).

The roles of various groups of PLP have not been investigated for these enzymes. The role of the hydroxyl group, as suggested by the computation study mentioned above (Section 6.4.), in preventing the overstabilization of I^•^ should be investigated. This can be performed by using 3-deoxyPLP (Scheme 5), which needs to be synthesized and might result in detection of corresponding elusive I^•^. In addition, the function of hydroxyl group was investigated by synthesizing 3-deoxy-3-fluoropyridoxamine 5′-phosphate (3-deoxy-3-fluoroPMP), pyridoxamine 5′-phosphate (PMP) analog, and employing it in the reaction of CDP-6-deoxy-l-*threo*-d-*glycero*-4-hexulose-3-dehydrase, a PMP/[2Fe–2S]-containing enzyme [100]. The related vitamin B_6_ in 5,6-LAM and 4,5-OAM, 3-deoxy-3-fluoroPLP (Scheme 5), could also be used to study the role of the hydroxyl group. Furthermore, there have been relevant studies using synthesized vitamin B_6_ analogs to unravel the role of those groups in related enzymes. For instance, the role of nitrogen atoms was investigated [101–103] in aspartate aminotransferase (AAT), alanine racemase (AR), and *O*-acetylserine sulfhydrase (OASS) using 1-deazapyridoxal 5′-phosphate (1-deazaPLP, Scheme 5) [104]. AR and OASS reconstituted with 1-deazaPLP show ~250-fold decrease in activity, while AAT shows >10^9^-fold decrease in activity [102]. The interaction between 5′-phosphate and the substrate is responsible for substrate specificity and even optimal catalytic efficiency in serine palmitoyltransferase [105]. Therefore, it would be interesting to study the role of these groups, if any, in 5,6-LAM and 4,5-OAM.

Preliminary study on capability of OraS of 4,5-LAM to form a complex with KamDE of 5,6-LAM [41] must be explored further. It might provide the information regarding the suicide inhibition observed with substrates in the reaction with the recombinant 5,6-LAM.

In a recent review, Wu and coworkers proposed the potentiality of 5,6-LAM as a biocatalyst [106] because it can accept at least three substrates. This flexibility gives rise to speculation that it might accept a number of substrates that produce building blocks, chiral 1,4- or 1,3-diamine, for biologically active compounds. For example, a noncisplatin type complex containing propane-1,3-diamine has been reported to have anticancer activity [107]. Oligoamines, containing diamine building blocks, effectively inhibit growth of human breast cancer cell lines in culture [108]. 5,6-LAM may potentially be employed to synthesize various chiral diamine building blocks and eventually access relevant oligoamines having better activities. However, to explore the potential of 5,6-LAM as biocatalyst, we need to focus initially on two aspects: first, the binding interactions of substrate, intermediates, and product with the amino acid residues, and second, the stereochemistry involved in the reaction to successfully design the substrate for the desired product. The information about binding interactions might be obtained by performing meaningful site-directed mutagenesis as described above while the strategies to address the aspects related to stereochemistry are discussed below. Enlightened with this information and followed by using the strategy of two-component substrate mimic (see below), one would be able to unravel the potential of 5,6-LAM as biocatalyst. Although absolute configuration of the 3,5-diaminohexanoic acid produced in the 5,6-LAM reaction with β-lysine was determined to be (3*S*,5*S*) [109] long ago, the stereospecificities of the other two substrates are yet unknown. Recently, it was shown that the product from *E. coli* 2,3-LAM and clostridial 2,3-LAM are (*R*)-β-lysine and the enantiomer, respectively [110]. Therefore, it would be interesting to study the stereochemistry of the reaction with 5,6-LAM. d/l-Lysine has only one chiral centre at C2 while l-β-lysine has it at C3. However, for 5,6-LAM the reaction involves C6 and C5. There are two possibilities of hydrogen abstraction at C5. It can abstract either 5-*pro-S* or 5-*pro-R* hydrogen atom leading to 5*S* or 5*R* isomers respectively. Isolation of the product and assignment of the configuration at C5 would furnish the information about the stereospecifities, if any, of the reaction. In another strategy, the introduction of the chiral centre at C5 of lysine would allow an investigation of the nature of hydrogen abstraction. 5-Fluorolysine, in which a fluorine atom substitutes one of the two hydrogen atoms at C5, will produce the desired chiral centre at C5. As mentioned above (Section 6.4.), 5-fluorolysine has the potential to accumulate the corresponding substrate radical in the steady state [99]. This analog has four different diastereomers, which can be separated by chemical modification and chiral chromatography [111]. By performing EPR experiments of the reaction mixtures of above four diastereomers as substrates of 5,6-LAM, we shall be able to find out the structure of the radical intermediates and know which hydrogen atom in between 5-*pro-S* and 5-*pro-R* is abstracted.

**Scheme 6.**
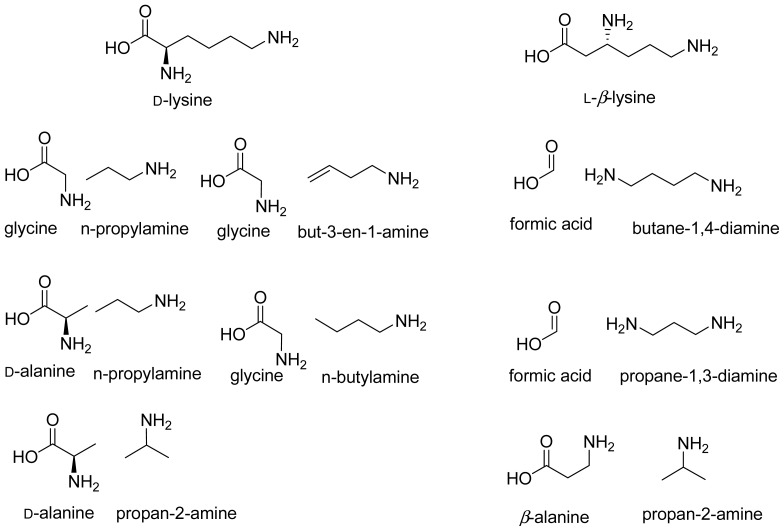
Two-component mimics of substrates.

2,3-LAM, an enzyme with high substrate-specificity for l-lysine, shows very low activity with l-alanine and l-2-aminobutyrate. The primary amines such as ethylamine and methylamine stimulate reactions of l-alanine and l-2-aminobutyrate by mimicking the active site binding and stabilizing the transition states. It was found that binding of l-alanine and ethylamine combination, as well as l-2-aminobutyrate and methylamine combination simulate the binding of l-lysine most efficiently and react to form β-alanine and 3-aminobutyrare, respectively [112]. There are several patents on the engineering of 2,3-LAM to 2,3-alanine mutase and subsequent use of it for the production of 3-hydroxypropionate, which is a precursor for polymers, adhesives and other useful chemicals [106]. Following the concept of 2,3-LAM, one can design many probable combinations of two-component mimic (Scheme 6) of the substrates of 5,6-LAM.

Considering the fact that combinations containing one carbon less with respect to the substrate produced the best result in the reaction of 2,3-LAM, we believe that the combination of *n*-propylamine and glycine would be the most promising. The combination of glycine and propylamines is a potential combination to produce chiral propylamine. A combination of ethylamine and d-alanine would also mimic the substrate. However, this combination is irrelevant for application purpose as the 1,2-shift of amino group will produce ethylamine itself. As 5,6-LAM is more flexible than 2,3-LAM, combinations, such as d-alanine and propylamines, having six carbon atoms are also promising. Similarly, combinations of glycine and butylamines, and formate/formic acid and diamines are also promising. These combinations as well as some other probable combinations are shown in Scheme 6.

## Figures and Tables

**Table 1.** Steady-state kinetic properties.

Enzyme	Substrate	*K*_m_ (mM)	*k*_cat_/*K*_m_ (M^−1^ s^−1^)	Reference
5,6-LAM	d-Lysine	20.0	6.3 ×10^2^	[24,42]
5,6-LAM	l-β-Lysine	8.7	4.8 ×10^2^	[24,42]
5,6-LAM	l-Lysine	20.0	0.4	[24,42]
4,5-OAM	d-Ornithine	0.2	15.2 ×10^3^	[43]
